# Carbon Nanotube Length Governs the Viscoelasticity and Permeability of Buckypaper

**DOI:** 10.3390/polym9040115

**Published:** 2017-03-23

**Authors:** Zhiqiang Shen, Magnus Röding, Martin Kröger, Ying Li

**Affiliations:** 1 Department of Mechanical Engineering, University of Connecticut, Storrs, CT 06269, USA; zhiqiang.shen@uconn.edu; 2 RISE Bioscience and Materials, Box 5401, 402 29 Göteborg, Sweden; magnus.roding@ri.se; 3 School of Energy and Resources, UCL Australia, University College London, Adelaide SA 5000, Australia; 4 Polymer Physics, Department of Materials, ETH Zürich, CH-8093 Zurich, Switzerland; 5 Institute of Materials Science, University of Connecticut, Storrs, CT 06269, USA

**Keywords:** buckypaper, carbon nanotube, viscoelasticity, permeability, molecular dynamics

## Abstract

The effects of carbon nanotube (CNT) length on the viscoelasticity and permeability of buckypaper, composed of (5,5) single-walled CNTs (SWCNTs), are systematically explored through large-scale coarse-grained molecular dynamics simulations. The SWCNT length is found to have a pronounced impact on the structure of buckypapers. When the SWCNTs are short, they are found to form short bundles and to be tightly packed, exhibit high density and small pores, while long SWCNTs are entangled together at a low density accompanied by large pores. These structure variations contribute to distinct performances in the viscoelasticity of buckypapers. The energy dissipation for buckypapers with long SWCNTs under cyclic shear loading is dominated by the attachment and detachment between SWCNTs through a zipping-unzipping mechanism. Thus, the viscoelastic characteristics of buckypapers, such as storage and loss moduli, demonstrate frequency- and temperature-independent behaviors. In contrast, the sliding-friction mechanism controls the energy dissipation between short SWCNTs when the buckypaper is under loading and unloading processes. Friction between short SWCNTs monotonically increases with rising length of SWCNTs and temperature. Therefore, the tanδ, defined as the ratio of the loss modulus over the storage modulus, of buckypaper with short SWCNTs also increases with the increment of temperature or SWCNT length, before the SWCNTs are entangled together. The permeability of buckypapers is further investigated by studying the diffusion of structureless particles within buckypapers, denoted by the obstruction factor (β). It is found to be linearly dependent on the volume fraction of SWCNTs, signifying a mass-dominated permeability, regardless of the structure variations induced by different SWCNT lengths. The present study provides a comprehensive picture of the structure-property relationship for buckypapers composed of SWCNTs. The methodology could be used for designing multifunctional buckypaper-based devices.

## 1. Introduction

Carbon nanotubes (CNTs) with persistence lengths $L_{p}$ of the order of several microns are one of the most attractive nanomaterials due to their exceptional mechanical, thermal and electrical properties [1,2,3,4,5]. Specifically, the Young’s modulus and breaking strength of individual CNTs are experimentally found to be up to 950 GPa and 52 GPa [6,7], respectively. Therefore, CNTs have been widely recognized as one of the strongest materials. According to their outstanding properties, individual CNTs have been adopted to be building blocks of various thermally-stable and viscoelastic materials, such as aligned sparse CNT arrays [8], films [9] and dense CNT brushes [10]. CNT buckypaper is one of these assemblies, characterized by the randomly entangled CNT networks. Buckypapers are typically fabricated by firstly purifying the individual CNTs and then dispersing them into a suitable solvent. Afterwards, the suspension can be further membrane filtered under positive and negative pressure, yielding a stable film. During this process, the CNTs exhibit the strong tendency to aggregate due to their long range van der Waals (vdW) interactions, forming either bundle-dominated or entanglement-dominated CNT networks [11,12]. The fabricated buckypaper demonstrates low density 0.05–0.4 g/cm${}^{3}$ and a high porosity of 0.8–0.9 [13]. The Young’s modulus and breaking strength of single-walled CNTs (SWCNTs) buckypapers are up to 4.2 GPa and 33 MPa, respectively [14,15,16,17]. Their mechanical properties could be further tuned by adjusting the density and the CNT diameter [18,19]. All of these properties make buckypapers attractive nanomaterials with many potential applications, such as sensors, actuators, filtration and distillation devices.

Here, we take the application of filtration as an example. The high porosity of buckypapers devotes the fast mass transport for water, gas or other solutions. Combining with their excellent mechanical properties, buckypapers are considered to have a great potential in filtration, overcoming the shortness of polymeric membranes in fouling, short service lifetimes and low chemical selectivity [20,21,22]. For example, considering the specific properties of SWCNT buckypapers, such as small diameter, high surface area and large porosity, Annal et al. experimentally demonstrated that the SWCNT buckypapers could be used to effectively remove bacterial and viral pathogens from water [23]. Decorating the purified multi-walled CNTs (MWCNTs) buckypapers with a hydrophilic group, Yang et al. proved that MWCNTs buckypapers had an excellent removal of humic acid from water [24]. It was also suggested that buckypapers could be applied for desalination and gas separation due to their porosity [25,26]. Besides, the permeability of buckypapers also plays an important role in composite manufacturing. Wang et al. reported that by infiltrating epoxy resin along the thickness direction of SWCNTs buckypaper, the SWCNTs network could be converted into the nanocomposites of buckypaper/resin with much higher storage modulus than neat resin [27]. Liu et al. also found that the laminar fracture toughness of a composite laminate can be significantly improved by incorporating the buckypaper into the middle interface of the laminate [16]. Meng et al. adopted the buckypaper as a template to produce CNT-polyaniline composite with higher specific capacitance, lower resistivity and higher stability under different current loads, which have promising applications in the field of energy storage devices [28].

Along with many potential applications of buckypapers, many works have been done to understand their mechanical properties and underlying physical mechanisms at the molecular level. For instance, the viscoelasticity of buckypapers is demonstrated to be frequency and temperature independent by experiments [29] and simulations [30]. The attachment and detachment between different CNTs through the zipping-unzipping mechanism have been identified to play the most important role in this process [29,30]. With the help of molecular simulations, it is found that both the entanglement and bundling mechanisms play important roles in the structural and mechanical properties of buckypapers. Due to the competition between these two mechanism, 50 wt % is found to be the optimal content of (8-8)-(12,12) double-walled CNTs (DWCNTs) embedded within (5,5) SWCNTs, yielding the largest value of Young’s modulus for their mixtures [12]. Although extensive works have been done to explore the mechanical properties of buckypapers or CNT networks [31,32,33,34,35], few of them have explored the CNT length effect. Recently, Chen et al. investigated the length effect of SWCNT buckypapers on their viscoelasticity [36]. However, the smallest length considered was L=100 nm in their works, which does not cover the whole spectrum of available SWCNT lengths. Thus, the role played by the SWCNT length might not be revealed in these simulations. Besides, the permeability of buckypaper is determined by the pore shape, pore size, film thickness and tortuosity of flow path [22,37,38]. It will be of great interest to control the permeability of liquid or gas through the buckypapers. For example, the permeability of buckypapers plays an important role in resin infiltration and void formation, which further affect the mechanical properties of buckypaper composites [37]. However, the underlying mechanism governing the permeability of buckypaper is difficult to confirm due to multiple parameters and their interactions, which affects the microstructure of buckypaper during the fabrication process.

Here, we investigate the influence of SWCNT length on the viscoelasticity and permeability of buckypapers. Particularly, the microstructure changes of buckypapers are related to different SWCNT lengths, which will be further correlated to their viscoelasticity and permeability. This work is organized as follows. The simulation models and methodologies for calculating viscoelasticity and permeability of buckypapers are given in Section 2. In Section 3, the structural and mechanical properties of buckypapers are presented as functions of SWCNT length. Interestingly, the microstructure of buckypaper is found to be significantly affected by the SWCNT length. In turn, the viscoelasticity and permeability of buckypaper are also influenced by the SWCNT length, due to microstructural changes. The viscoelasticity of buckypaper is found to be governed by sliding-friction and zipping-unzipping mechanisms for short and long SWCNTs, respectively. Moreover, the permeability of buckypaper is found to follow a scaling law with the volume fraction of SWCNTs, signaling a mass-dominated permeability behavior. In Section 4, the relationship between the microstructure and viscoelasticity of buckypapers is discussed in detail. Concluding remarks are given in Section 5.

## 2. Computational Model and Methodology

### 2.1. Coarse-Grained Model for SWCNTs

**Figure 1:**
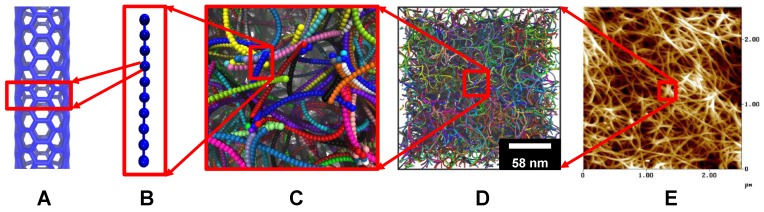
Configurations of SWCNTs at different length scales: (**A**) all-atomic model; (**B**) coarse-grained model; (**C**) entangled SWCNTs; (**D**) microstructure of SWCNT buckypaper; and (**E**) atom force microscope (AFM) image of SWCNT buckypaper. In (B), each coarse-grained bead represents a SWCNT segment in the corresponding all-atomic model. Individual SWCNTs are denoted by different colors in (C,D). The AFM image is adapted from [27] with permission.

To explore the SWCNT length effect, CNT assemblies with different SWCNT lengths are built. Considering the long-range vdW interaction and relative large length of CNTs in their assemblies, a coarse-grained model for (5,5) SWCNTs developed by Buehler [39] is adopted in our study. In this model, each SWCNT has been discretized into a multi-bead chain. All of the beads on this chain are sequentially connected by harmonic springs, where an angle potential is applied among three consecutive beads to ensure the stiffness of SWCNT. Non-bonded beads interact with each other through pair-wise potentials, capturing long-range vdW interactions. Therefore, each bead in this coarse-grained model represents a segment of SWCNT in the all-atomic model, as shown in Figure 1. Following a “finer-train-coarser” approach, the coarse-grained model is calibrated through the uniaxial tension, cantilever bending and adhesion tests on the all-atomic SWCNT model [39]. Thus, the Young’s modulus, bending stiffness and interatomic friction between SWCNTs can be correctly reproduced through the coarse-grained model. The total energy of the SWCNT can be expressed as $U_{\mathrm{system}}=U_{\mathrm{bond}}\left(b\right)+U_{\mathrm{angle}}\left(\theta \right)+U_{\mathrm{vdW}}\left(r\right)$. Specifically, $U_{\mathrm{bond}}\left(b\right)=\frac{k_{b}}{2}{\left(b-b_{0}\right)}^{2}$, where $k_{b}=1000$ kcal mol${}^{-1}$Å${}^{-2}$ is the bond stretch coefficient, which is chosen to reproduce the Young’s modulus of SWCNT. *b* is the bond distance, and $b_{0}=$ 10 Å is the equilibrium bond distance. $U_{\mathrm{angle}}\left(\theta \right)=\frac{k_{a}}{2}{\left(\theta -\theta_{0}\right)}^{2}$, where $k_{a}=$ 14,300 kcal mol${}^{-1}$ rad${}^{-2}$ is the bending constant and determined by the bending stiffness of SWCNT. θ is the angle formed by three beads in sequence, and $\theta_{0}=180^{\circ }$ is the equilibrium angle. The Lennard–Jones 12:6 (LJ) potential is chosen to represent the long-range vdW interaction. $U_{\mathrm{vdW}}\left(r\right)=4\epsilon \left[{\left(\sigma /r\right)}^{12}-{\left(\sigma /r\right)}^{6}\right]$, with σ=9.35Å as the distance parameter, and ϵ=15.10 kcal mol${}^{-1}$ is the energy well depth at equilibrium; *r* is the distance between two beads. Thus, a coarse-grained SWCNT is characterized by six parameters: $k_{b}$, $b_{0}$, $k_{a}$, $\theta_{0}$, ϵ, σ, as well as its length *L*. The system composed of SWCNTs is further characterized by temperature, pressure and total amount of SWCNTs. The above coarse-grained model has been successfully used to reproduce the microstructure and mechanical properties of CNT buckypapers [12,40], viscoelasticity of CNT buckypapers [30,36], energy dissipation of CNT buckypaper under impact [34] and mechanical properties of CNT yarns [41,42,43].

### 2.2. Initial Configuration of SWCNT Buckypapers

The structure of molecular systems is of great importance to determine their performance. For the CNT buckypapers in our simulations, their ultimate structures are sensitive to the initial configurations. To obtain randomly entangled and isotropic in silico buckypapers, a self-avoiding random walk approach is adopted to generate the initial configuration. As we reported in our previous works [12], this kind of initial configuration can be successfully used as the pre-equilibrated configuration. Then, the generated structure is further relaxed through a combined molecular dynamics (MD)/Monte Carlo (MC) method, according to the bond swap algorithm [44]. After the step of MD/MC, the system is further relaxed under isothermal-isobaric ensemble in two steps. First, a high temperature (T=1000 K and 1 atm) condition is applied to facilitate a rapid relaxation. After 100 ns (or $10^{7}$ time steps, the time step in all of our simulations is taken to be 10 fs) under this high temperature, the system is gradually annealed to the normal room temperature (T=300 K and 1 atm pressure) within 40 ns and further relaxed at T=300 K for 40 ns. Finally, the 3D highway networks can be produced for long SWCNTs (cf. Figure 1), as the one given in experiments. Following a similar protocol, the obtained CNT buckypapers have been tested to be able to reproduce the pore size distribution and density in experiments in our previous simulations [12]. Details about how to generate the initial configuration are given in [12]. To systematically explore the SWCNT length effect on the viscoelasticity of buckypapers and their permeability, the individual SWCNT length in our simulations systematically varies from L=2nm to L=1999nm. The total number of beads in all studied systems is fixed at N= 400,000, corresponding to a total SWCNT length of $\left(N-1\right)b_{0}≃0.4$ mm and a total mass of $Nm_{0}=781.3$ MDa, where $m_{0}=1953.23$ g/mol is the estimated mass represented by a single bead. Under these conditions, the relaxed simulation box length exceeds the radius of gyration of the longest individual SWCNT (L=1999 nm) by a factor of two. Note that the short SWCNTs might not be able to form networks, while we still denote the relaxed structures as buckypaper for simplicity. Here, we should emphasize that the persistence length $L_{p}$ of the individual (5,5) SWCNT is about 10 µm [39,45,46]. According to the worm-like chain model [47], using the measured values for mean squared end-to-end distance and gyration radius, the $L_{p}$ of the SWCNT networks are 10.6, 22.7, 0.0180, 0.0071, 0.0036, 0.0029 and 0.0028 µm for L= 2, 9, 49, 99, 499, 999 and 1999 nm, respectively. Therefore, we effectively capture the whole $L/L_{p}$ range from stiff to flexible, while the intrinsic bending stiffness of SWCNT is constant.

### 2.3. Non-Equilibrium Molecular Dynamics Simulations

The viscoelastic properties of SWCNT buckypapers are investigated through non-equilibrium molecular dynamics (NEMD) simulations based on the GSLLOD algorithm [48], which is derived from rigorous statistical-mechanical principles, being proved to be equivalent to Newton’s equations of motion for shear flow [49]. Coupled with a Nosé–Hover thermostat [50,51], it have been demonstrated to generate the desired velocity gradient and the correct production of work by stresses for all forms of homogeneous flow in the canonical ensemble [49] by solving the following set of equations:

(1) $$\begin{array}{ccc} {\dot{q}}_{i} & = & \frac{p_{i}}{m_{i}}+q_{i}\cdot \nabla u, \end{array}$$

(2) $$\begin{array}{ccc} {\dot{p}}_{i} & = & F_{i}-p_{i}\cdot \Delta u-m_{i}q_{i}\cdot \nabla u\cdot \nabla u-\frac{p_{\xi }}{Q}p_{i}, \end{array}$$

(3) $$\begin{array}{ccc} \dot{\xi } & = & \frac{p_{\xi }}{Q}, \end{array}$$

(4) $$\begin{array}{ccc} {\dot{p}}_{\xi } & = & \sum_{i=1}^{N}\frac{p_{i}^{2}}{m_{i}}-dNk_{B}T \end{array}$$

where the dot represents a derivative with respect to time, ∇ denotes a spatial gradient and ∇u the imposed macroscopic velocity gradient evaluated at $q_{i}$, while $q_{i}$, $p_{i}$, $m_{i}$ and $F_{i}$ are the position vector, momentum vector, mass and force vector of bead *i*, respectively. d=3 is the space dimensionality; *N* is the total number of beads; ξ and $p_{\xi }$ are the coordination and momentum-like variables of the Nosé–Hoover thermostat. $Q=dNk_{B}T\lambda^{2}$ is the thermostat mass parameter, where λ is the characteristic relaxation time, which is taken as 2ps in our simulations. $k_{B}$ is the Boltzmann constant, and *T* represents the temperature. Lees–Edwards periodic boundary conditions [52] are applied during the NEMD simulations, that is for a bead crossing the moving boundary, not only its position is shifted, but also its velocity is adjusted by adding the prescribed velocity difference between lower and upper boundaries. All of the simulations are done via LAMMPS software supplied by Sandia National Laboratories [53]. Visualizations have been operated by using the VMD package [54].

### 2.4. Dynamic Viscosities

According to the linear viscoelasticity theory [55], there is a phase shift between an input shear strain ∇u and the output shear stress, as expressed below:

(5) $$\gamma =\gamma_{0}\sin\omega t,\;\tau_{xy}=\tau_{0}\sin\left(\omega t+\delta \right)$$

where γ is the input strain of the oscillatory shear flow, $\gamma_{0}$ and ω represent the strain magnitude and frequency, respectively, and $\tau_{xy}$ is the xy-component of the Irving–Kirkwood stress [56,57,58]. $\tau_{0}$ and δ represent the stress magnitude and phase shift, respectively; *t* denotes time. Then, the frequency-dependent storage modulus $G^{\prime }$ and loss modulus $G^{\prime \prime }$ are estimated via:

(6) $$G^{\prime }\left(\omega \right)=\frac{\tau_{0}}{\gamma_{0}}\cos\delta ,\;G^{\prime \prime }\left(\omega \right)=\frac{\tau_{0}}{\gamma_{0}}\sin\delta$$

Their ratio $\tan\delta =G^{\prime \prime }/G^{\prime }$ defines the loss tangent. The value of the phase shift δ for a viscoelastic material resides within the extreme values for a pure elastic material (δ=0) and a Newtonian fluid (δ=π/2). The $\tau_{0}$, $\gamma_{0}$ and δ for the investigated systems we obtained by extracting data of the input strain and output stress and fitting them using least squares. The storage modulus $G^{\prime }$ and loss modulus $G^{\prime \prime }$ were then calculated via Equation (6). Other linear viscoelastic quantities, such as the dynamic viscosity $\eta^{\prime }\equiv G^{\prime \prime }/\omega$, are then available, as well [55].

### 2.5. Permeability

The permeability of buckypapers is characterized by the diffusivity of gas molecules or a liquid through buckypapers. For efficiently simulating diffusion, each buckypaper is discretized as a binary, solid-void voxel matrix, characterized by its linear voxel size $l_{v}$. All so-called 1-voxels whose centers reside within the interior of any of the locally cylindrical SWCNTs are assigned to have a value of unity; remaining ones are set to zero and are denoted as 0-voxels. To be specific, we are interested in results for the effective self-diffusion coefficient of structureless tracer particles, $D_{\mathrm{eff}}$, relative to their diffusion coefficient within the bulk, $D_{0}$; the ratio is known as the obstruction factor $\beta =D_{\mathrm{eff}}/D_{0}\in \left[0,1\right]$. Two simulation methods are employed that gave basically identical results:

(I) We used the lattice Boltzmann method, a numerical framework for solving partial differential equations based on simulating local particle populations, to solve the diffusion equation:

(7) $$\frac{\partial c\left(x,t\right)}{\partial t}-\nabla \cdot \left[D\left(x\right)\nabla c\left(x,t\right)\right]=0,$$

for a concentration *c* of a diffusing species with local diffusion coefficient D(x), equal to $D_{0}$ in void voxels and 0 in solid voxels. The lattice Boltzmann method used here is a two relaxation-time method with a non-constant scalar diffusion coefficient [59]. By applying a relative concentration difference Δc over a length Δx in the *x*-direction across the structure, the average diffusion flux *j* (averaged over the simulation domain) is computed from the steady-state solution. The effective diffusion coefficient $D_{\mathrm{eff}}$ is then computed from Fick’s law,

(8) $$\beta =\frac{D_{\mathrm{eff}}}{D_{0}}=-\frac{j\Delta x}{D_{0}\Delta c},$$

In the other two directions, mirror boundary conditions are used. For more details on the method, see [19,60,61].

(II) We simulated a Monte Carlo ensemble of discrete random walks with *n* steps of step length $l_{v}$, whose nodes reside on the centers of the 0-voxels because each walk is assumed to get reflected during collision with a 1-voxel. To this end, each walk starts randomly from one of the available 0-voxels with equal probability. If a new random step were to coincide with the position of a 1-voxel, this step is simply forbidden, while the *n*-step counter proceeds. Periodic boundary conditions are used. The obstruction factor is then obtained from the mean square end-to-end distances *R* of the ensemble of walks, $\beta =\langle R^{2}\rangle /nl_{v}^{2}$, because $\langle R^{2}\rangle =nl_{v}^{2}$ for the random walk in the absence of obstacles and because the diffusion coefficient can be expressed in terms of the mean square displacement of a tracer particle. Its trace actually coincides with the generated walk, and the result is, as already mentioned, independent on $l_{v}$, if $l_{v}$ is smaller than the CNT radius and also independent of *n* if $\sqrt{n}l_{v}$ is taken large compared with the largest pore size divided by voxel size, which is computationally unproblematic. We used an ensemble of 10,000 walks, where each walk had at least C= 150 collisions (this is realized using n≈C/ϕ steps, where ϕ denotes the volume fraction of the filled CNTs, here the number of 1-voxels divided by all voxels). To improve the efficiency, one can simulate two walks using the same seed value, one on the partially filled grid, one on an empty grid, and calculate β from the ratio of mean square end-to-end distances, instead of assuming that $\langle R^{2}\rangle =nl_{v}^{2}$ perfectly holds for a finite ensemble of undisturbed random walks.

For both methods, we find the measured β to be independent of voxel size as long as $l_{v}$ is smaller or still comparable with the SWCNT radius (≈0.5 nm). We also find the choice of $l_{v}=$ 0.2 nm, for which results will be presented, to be a good compromise between numerical efficiency and precision: results do not change upon further decreasing $l_{v}$ to the expense of a larger memory requirement. The effect of the size of the tracer particle can be mimicked by choosing $l_{v}$ larger than the SWCNT radius.

### 2.6. Pore Size Distribution and Entanglement Analysis

Pore sizes and entanglements between SWCNTs are important structural properties for buckypaper in two different aspects. The diffusion of gas/liquid in buckypapers should be determined by factors like pore shape, pore size and the tortuosity of the flow path. On the other hand, inter-tube entanglements can greatly affect the loading transfer efficiency among the discontinuous CNTs [62]. In addition, the viscoelasticity of buckypaper is considered to be highly related to the number of inter-tube entanglements between CNTs [30,36]. To investigate their relation, the pore size and entanglement information will be explored in this study. The pore size in SWCNT buckypapers we analyze through the Euclidean distance map (EDM) method [63]. As we did in computing the permeability, the buckypaper is discretized into a binary voxel matrix composed of 0-voxels and 1-voxels. A fitting sphere is defined and calculated for each 0-voxel according to the smallest distance to the 1-voxels. The sphere radii are collected to compute the pore size distribution. The result is independent of the voxel size as long as it is sufficiently small [12]. To extract the entanglement information, a geometric algorithm, the Z1code [64,65], is adopted. In the Z1 code, each multi-bead SWCNT is mapped onto an infinitely thin chain that permanently shares the positions of the terminal ends with the original SWCNT and is constructed to represent the shortest path between the ends, taking into account the uncrossability of the chains. During this procedure, a fluctuating number of nodes is introduced until the total length of the disconnected path has reached a global minimum. The resulting network is denoted as a network of primitive paths (PP) and is characterized by the number of interior kinks (entanglements) denoted as *Z*. Details about the Z1 code are described in [64,65]. It has been successfully applied to various polymeric systems to analyze their entanglement network [66,67,68], including CNT networks [12,30,40].

## 3. Results

### 3.1. Structural Properties of Buckypaper

**Figure 2:**
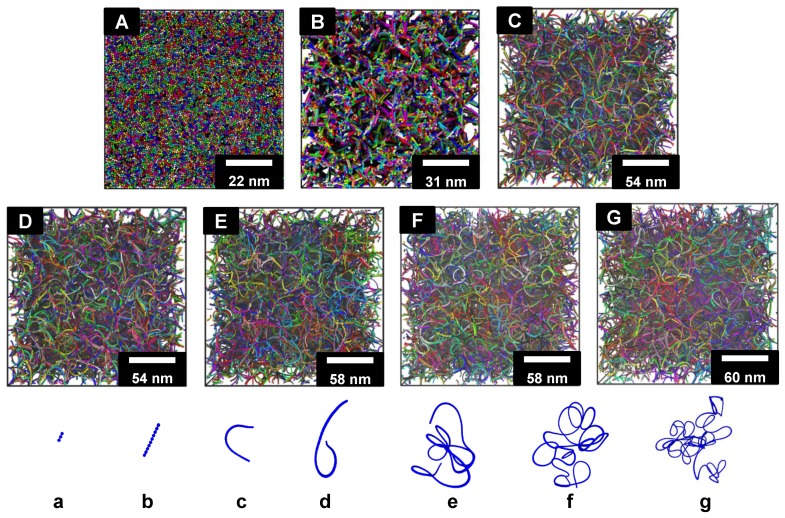
Fully-relaxed configurations of buckypaper with different SWCNT lengths. (**A**–**G**) represent L=2nm, L=9nm, L=49nm, L=99nm, L=499nm, L=999nm and L=1999nm, respectively. Different colors represent different SWCNTs. (**a**–**g**) are the individual SWCNTs in the corresponding microstructure of (A–G), denoting the configuration of a single SWCNT, respectively. SWCNTs in (a–g) are plotted at different scales.

The fully-relaxed microstructure of SWCNT buckypaper is determined by the competition between the long-range vdW attraction (inter-tube adhesion energy) and bending energy penalty due to the high stiffness of individual CNTs. Under normal conditions, long SWCNTs tend to self-assemble together and bend to increase their contact length, due to the strong inter-tube adhesion. As shown in Figure 2, the microstructure of buckypaper highly depends on the length of SWCNTs. Extremely short SWCNTs (L=2 nm) tend to mix together due to their vdW interactions, resulting in a tightly-packed structure without visible pores. Slightly longer SWCNTs with L=9nm align together due to inter-tube adhesion, forming bundle-like structures to minimize the bending penalty. These bundles orient randomly to increase the configurational entropy, resulting in pores between them. If we inspect the individual configurations of SWCNTs (cf. Figure 2b), each of them is straight without any pronounced bending. Upon increasing the SWCNT length further, such as L=49 nm, the microstructure of buckypaper renders similarity to a 3D highway network, as demonstrated in Figure 2C. This network is characterized by a pronounced bending of individual SWCNTs, separated by pores. Very long SWCNTs (L=499 nm) can easily form ‘interwound’ structures due to the strong inter-tube adhesion energy [40], overcoming the bending penalty. In contrast, the short SWCNTs (L=9 nm) are straight due to the high bending energy penalty, which cannot be compensated by the inter-tube adhesion energy.

**Figure 3:**
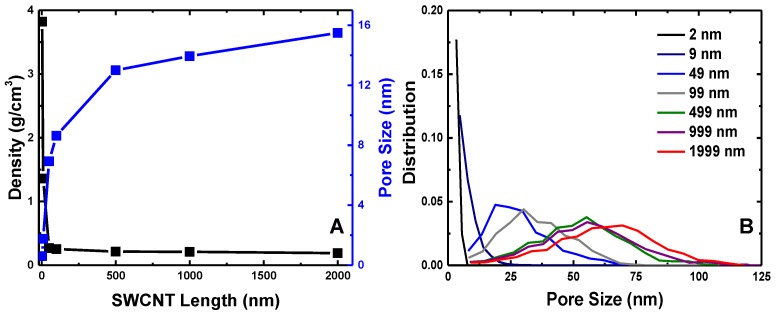
(**A**) Density (black) and average pore size (blue) of buckypaper as a function of SWCNT length *L*; (**B**) pore size distribution of SWCNT buckypapers with different *L* (specified in the legend).

To further evaluate the SWCNT length-dependent behaviors of buckypapers, we extract and compare their densities, average pore sizes and pore size distributions. As shown in Figure 3, the density of buckypaper reduces dramatically at about L= 49 nm, due to the tightly-packed microstructure of buckypapers with shorter *L*. For L>50 nm, the density of SWCNT buckypaper reaches a plateau exhibiting a density of 0.17 g/cm${}^{3}$, without any further visible change. This value of density falls within the range of experimental reported densities of CNT buckypapers, 0.05–0.4 g/cm${}^{3}$ [11,69,70]. In accord with the reduction of density, the average pore size of the buckypaper monotonically increases with increasing SWCNT length. The pore size increases dramatically with *L* up to L=500 nm, due to the changes of their microstructures (cf. Figure 2). Beyond the SWCNT length L=500 nm, the average pore size assumes a value of about 14–16 nm, in good agreement with experimental observations [71]. Moreover, the distribution of pore sizes for buckypaper with long SWCNTs is much wider than that of buckypaper with short SWCNTs, as demonstrated by Figure 3B, due to their different microstructures. Noteworthy, the pore size distributions of L=499 nm, L=999 nm and L=1999 nm are almost identical. While the short SWCNTs (L=2 nm and L=9 nm) lead to tightly-packed microstructures for buckypapers, with high density and a small pore size, the ‘interwound’ long SWCNTs result in 3D highway structures for buckypapers, characterized by low density and large pore sizes.

**Figure 4:**
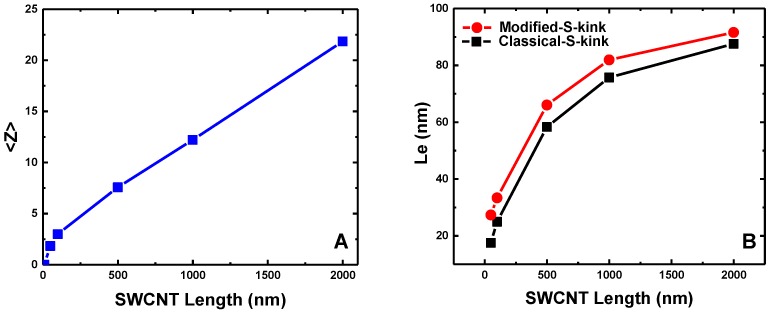
Entanglement analysis on buckypapers with different SWCNT lengths: (**A**) mean number of entanglements per SWCNT, 〈Z〉; and (**B**) entanglement length $L_{e}$ estimated by classical and modified S-kink estimators.

The entanglement networks of CNT buckypapers are further analyzed through the Z1 algorithm [64,65], as given in Figure 4. The mean number of inter-tube entanglements per SWCNT, 〈Z〉, is negligible and close to zero for L=2 nm and L=9 nm, consistent with the non-bending status of individual, short SWCNTs and a minimum number of bonds required to form a physical ‘knot’ [67]. Afterwards, 〈Z〉 linearly increases with increasing SWCNT length with an inverse slope of about 100 nm, indicating the SWCNT bending-facilitated entanglement behavior, affected by its semiflexibility. Using the number of inter-tube entanglements, we can estimate the entanglement length $L_{e}$ of (5,5) SWCNTs, which is defined as the averaged length between two adjacent entanglements. According to the previous study [67], $L_{e}$ can be estimated by equations of the ‘classical S-kink’ $L_{e}=L\left(L-b_{0}\right)/\left[\langle Z\rangle \left(L-b_{0}\right)+L\right]$ and the ‘modified S-kink’ $L_{e}=L/\langle Z\rangle$, which provide lower and upper bounds of the true entanglement length, respectively. The estimated $L_{e}$ increases rapidly with the chain length, as usual, and seems to reach $L_{e}\approx 100$ nm in the limit of infinitely long SWCNTs, as can be estimated from the inverse asymptotic slope in Figure 4A.

### 3.2. Viscoelasticity and Permeability

Having inspected the different microstructures of buckypapers with different SWCNT lengths, we turn to the study of their viscoelasticity and permeability. As demonstrated both in experiments [29,72] and simulations [30,36], the viscoelasticity of buckypaper composed of long CNTs is both temperature and frequency independent. Such a behavior is induced by the exceptional energy dissipation mechanism between CNTs. During cyclic loading and unloading, CNTs can attach and detach through a zipping-unzipping mechanism. Such a zipping-unzipping mechanism is highly sensitive to the amount of entanglements between different CNTs [30], occurring extremely quickly and stably as long as the loading-unloading frequency is not too high. However, the physical mechanisms for the viscoelasticity of buckypapers without inter-tube entanglements are not clear. Which role is played by the long-range vdW interaction under this nonequilibrium condition? In addition, the permeability of the membrane is determined by its pore shape and the pore size tortuosity of the flow path [22,37,38]. As previously mentioned, the pore size and their distribution in buckypapers can be greatly affected by the SWCNT length. In turn, the permeability of buckypaper can also be influenced by the SWCNT length. In the following part, we will attempt to explore the SWCNT length effects on the viscoelasticity and permeability of buckypapers.

**Figure 5:**
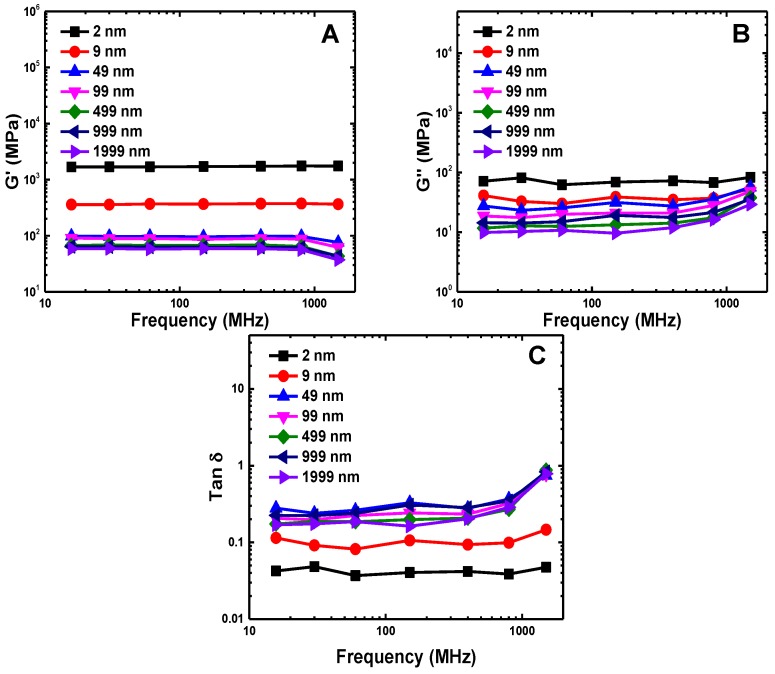
Viscoelasticity of buckypapers characterized by different SWCNT lengths (*L* values mentioned in the legends): (**A**) storage modulus $G^{\prime }$; (**B**) loss modulus $G^{\prime \prime }$; and (**C**) loss tangent $\tan\delta =G^{\prime \prime }/G^{\prime }$.

To systematically investigate the viscoelasticity of SWCNTs buckypaper, a series of simulations has been performed within a wide range of frequencies from 15.7MHz–1.5GHz. The strain amplitude in all of these simulations is chosen to be γ=0.03. The amplitude is large enough to avoid solely measuring the noise from thermal fluctuations and small enough to keep the deformation of buckypaper remaining within the region of linear response [30]. It is interesting to see that the storage $G^{\prime }$ and loss $G^{\prime \prime }$ moduli and the loss tangent tanδ are frequency independent, regardless of the SWCNT length, except for an extremely high frequency of 1.5GHz, as given in Figure 5. This renders the buckypaper to behave as a strong gel within this range of frequencies. We should also emphasize that within the frequency-independent region, both $G^{\prime }$ and $G^{\prime \prime }$ decrease with increasing SWCNT length, signaling the pronounced SWCNT length effect. In contrast, the loss tangent tanδ increases with SWCNT length increasing and tends to converge to a single value for L≥50 nm.

**Figure 6:**
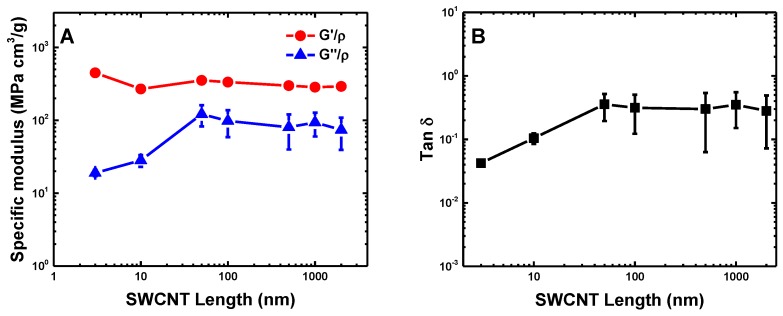
(**A**) Specific storage and loss moduli and (**B**) loss tangent of buckypapers as functions of SWCNT length. These values are taken from the frequency-independent regime.

The density of buckypapers has a great impact on their elastic and viscoelastic properties [12,36,72]. The large values of $G^{\prime }$ and $G^{\prime \prime }$ of SWCNT buckypapers might be induced by their relative high densities. To rule out the density effect and further explore the underlying physical mechanisms of energy dissipation during the cyclic shear process, we further compare the specific moduli $G^{\prime }/\rho$ and $G^{\prime \prime }/\rho$ under the influence of SWCNT length, as shown in Figure 6. It is noteworthy that $G^{\prime }/\rho$ slightly decreases with SWCNT length increasing and quickly approaches the plateau region. Thus, $G^{\prime }/\rho$ is insensitive to the SWCNT length. In comparison, $G^{\prime \prime }/\rho$ increases dramatically before L=100nm, after which it reaches a plateau regime. The loss tangent tanδ follows the same trend as the specific loss modulus $G^{\prime \prime }/\rho$. The different trends in the elastic (storage) and viscous (loss) moduli can be understood in the following two aspects. On the one hand, the stored elastic energy during the loading and unloading process of buckypapers is mainly determined by the packing density of SWCNT beads, which is proportional to the density, since all of these (5,5) SWCNTs have an identical Young’s modulus and bending stiffness. Therefore, the specific storage modulus $G^{\prime }/\rho$ is insensitive to the different SWCNT lengths. In the previous study [36], the bending and buckling of long SWCNTs are considered to be the reason for the decrement of storage modulus with increasing SWCNT length. However, the results given in Figure 6 indicate that the elastic energy stored during the cyclic loading can be determined by the long-range vdW interactions under the small shear amplitude, which is directly related to the packing density of SWCNT beads. On the other hand, the SWCNT length-dependent behaviors of $G^{\prime \prime }/\rho$ and tanδ indicate different energy dissipation mechanisms for buckypapers with short and long SWCNTs during the cyclic loading. For instance, the viscosity of buckypapers with long SWCNTs is mainly induced by the zipping-unzipping mechanisms [30], determined by the inter-tube entanglements between different SWCNTs. Therefore, both $G^{\prime \prime }/\rho$ and tanδ are insensitive to the SWCNT length. However, for short SWCNTs with length L=49nm, the inter-tube entanglements are almost negligible (cf. Figure 4). Thus, the zipping-unzipping mechanism cannot be used to explain the SWCNT length-dependent viscosity of buckypapers with short SWCNTs. This also signifies that another mechanism dominates the energy dissipation of buckypapers with short SWCNTs.

**Figure 7:**
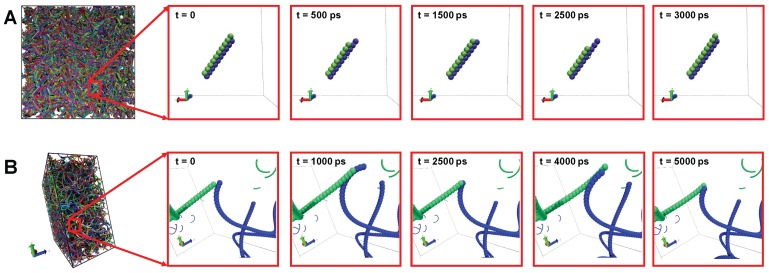
Different energy dissipation mechanisms for SWCNT buckypapers under cyclic shear: (**A**) sliding-friction mechanism between short SWCNTs (length L=9nm); (**B**) zipping-unzipping mechanism between long SWCNTs (length L=499nm).

To further explore the energy dissipation mechanisms for buckypaper under cyclic loading, we closely inspect their molecular configurations under the loading and unloading process, as presented in Figure 7. For short SWCNTs with length L=9nm, the bundles of SWCNTs keep intact during the shearing process. Since the individual SWCNT is confined inside its bundle by surrounding SWCNTs, it can only slide up and down during the cyclic loading to dissipate energy. Here, we name such an energy dissipation mechanism as the sliding-friction mechanism. Since the friction energy between SWCNTs within a bundle is proportional to the SWCNT length [73], the viscosity of buckypapers with short SWCNTs should also be length dependent, as revealed in Figure 6. Moreover, the energy dissipated during the sliding is smaller than the one induced by the SWCNT detachment [29] and will increase with the contact length, which is consistent with the increment of the loss tangent and specific modulus $G^{\prime \prime }/\rho$ before L=49nm. In contrast, the energy dissipation of long and entangled SWCNTs is dominated by the zipping and unzipping between entangled SWCNTs, as given in Figure 7. The dissipated energy during this process is not related to the total length of SWCNTs. Therefore, both $G^{\prime \prime }/\rho$ and tanδ are insensitive to the SWCNT length for buckypaper composed of long SWCNTs.

**Figure 8:**
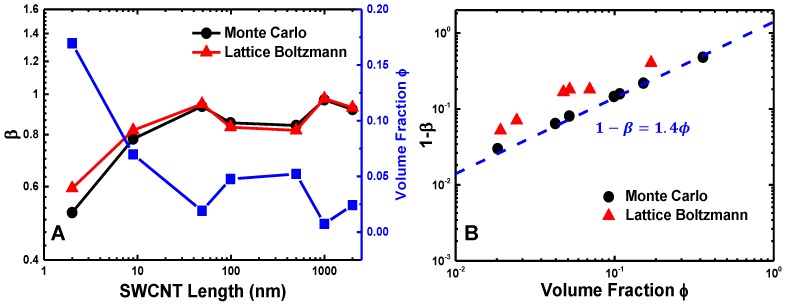
Obstruction factor of SWCNT buckypapers for characterizing permeability: (**A**) obstruction factor β as a function of SWCNT length; (**B**) obstruction factor 1−β as a function of the SWCNT volume fraction. I and II represent the lattice Boltzmann and discrete random walk methods, respectively. ‘Thin’ and ‘thick’ are two different SWCNT models described in Section 2.5.

The permeability of buckypaper is characterized through the obstruction factor β of structureless particles in the CNT networks. As shown in Figure 8, the lattice Boltzmann and discrete random walk Monte Carlo methods render almost identical results on β. The obstruction factor β monotonically increases with increasing SWCNT length and seems to reach a plateau regime when the SWCNT length exceeds L=49 nm. Such an observation is in good agreement with the average pore size and its distribution of SWCNT buckypapers given in Figure 3. To further explore the underlying mechanism affecting β, we inspect the relationship between the obstruction factor β and volume fraction ϕ of SWCNTs, which is defined as the fraction of one-voxels, i.e., the ratio between the volume of occupied voxels (one-voxels) and all voxels. It is surprising to find that the obstruction factor β decreases linearly with the volume fraction ϕ as β≈ 1–1.4 ϕ (Figure 8B). Note that a linear behavior and a prefactor of 1.5 has also been reported for the diffusion of ideal prolate, ellipsoidal particles through membranes [74]. Furthermore, the linear relationship might also suggest that the volume fraction should be the main factor to determine the obstruction factor β, irrelevant to the distinct microstructures of buckypapers of different SWCNT lengths.

### 3.3. Temperature Effect

**Figure 9:**
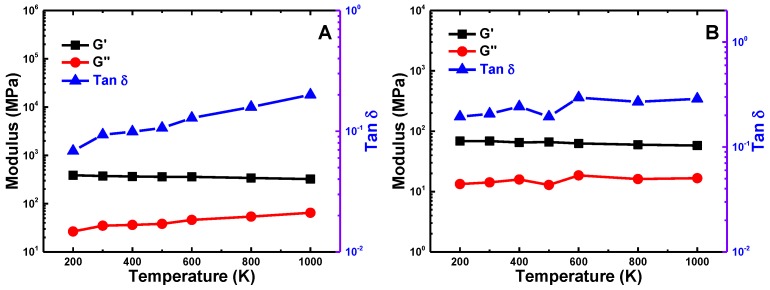
Temperature effect on the viscoelasticity of buckypapers with (**A**) short SWCNT (length L=9nm) and (**B**) long SWCNT (length L=499nn).

We further explore the possible temperature effects on the viscoelasticity of SWCNT buckypapers. Two typical SWCNT lengths are considered: L=9nm and L=499nm, which represent the sliding-friction and zipping-unzipping mechanisms, respectively. The frequency of the cyclic shearing is taken to be 400MHz. As given in Figure 9, $G^{\prime }$, $G^{\prime \prime }$ and tanδ of the buckypaper with long SWCNTs (L=499nm) are temperature independent, which is consistent with previous experimental [29] and computational [30] studies. However, for buckypaper with short SWCNTs (L=9nm), the loss modulus $G^{\prime \prime }$ and loss tangent tanδ monotonically increase with increasing temperature. On the contrary, the storage modulus $G^{\prime }$ is insensitive to the temperature change. As previously mentioned, the viscosity of buckypaper with short SWCNTs is induced by the sliding-friction mechanism. With the temperature increasing, the individual SWCNTs can easily slide further with high kinetic energies and escape from their bundle assemblies. Therefore, the dissipated friction energy, as well as $G^{\prime \prime }$ and tanδ, linearly increase with the increment of temperature. Since the packing density, Young’s modulus and bending stiffness of SWCNTs are insensitive to temperature, the stored elastic energy and storage modulus $G^{\prime }$ demonstrate temperature-independent behaviors.

## 4. Discussion

Our simulation results provide insights into the structure-property relationship for the SWCNT buckypapers, which might be generalized to other fibril networks. CNT is considered to be a semiflexible polymer with a large persistent length, which is longer than 9 µm [39,45,46]. As demonstrated in our previous study, the microstructure of CNT buckypaper is determined by the competition between the inter-tube binding energy ϑ and bending stiffness EI. Both the pore size and entanglement length are found to be dependent on the ratio $\sqrt{EI/\vartheta }$. Specifically, when $\sqrt{EI/\vartheta }<40$ nm, the inter-tube binding energy ϑ is the dominating factor, resulting in well-entangled CNT networks; while, if $\sqrt{EI/\vartheta }>40$ nm, the bending energy plays the dominating role, and the CNTs are bundled together in their network. For the given type of CNTs, such as (5,5) SWCNT, the configuration of individual SWCNTs and the microstructure of their networks can also be explained by the competition between the inter-tube binding energy and bending energy. When the SWCNT is short, the contact length between different SWCNTs is limited. Thus, the inter-tube adhesion energy is much smaller than the bending energy. To minimize the free energy of the system, the short SWCNTs tend to bundle together without obvious bending, as shown in Figure 2. When the length of SWCNT is long enough, the bending energy barrier can be compensated and defeated by the inter-tube adhesion energy. Thus, SWCNTs can be more easily bent and form entangled networks. In the meantime, the configuration of individual SWCNTs changes from the straight (L=2,9nm) to interwound (L=499,999,1999nm) structures. From the entanglement analysis, the entanglement length for (5,5) SWCNT is about 100 nm, which suggests that the transition from the CNT-bundled microstructure to CNT-entangled structure should occur around this length.

**Figure 10:**
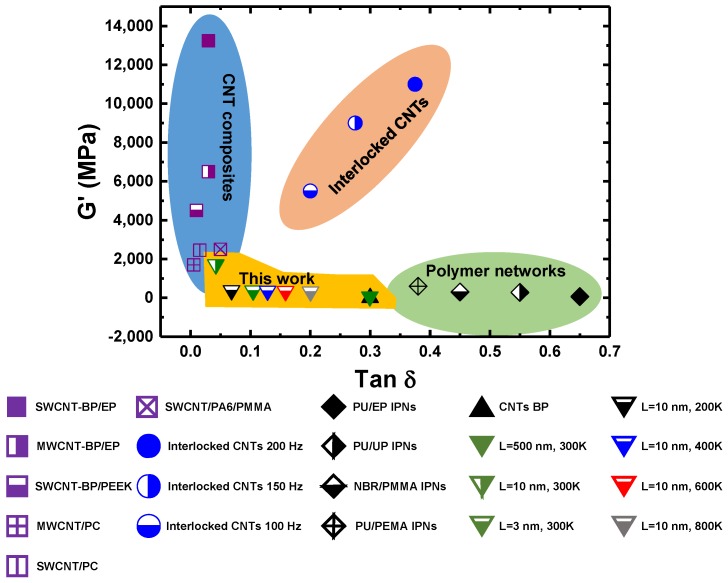
Viscoelasticity of buckypapers with different SWCNT lengths, in comparison with the experimental reported values of inter-penetrating polymer networks (IPNs), CNT composites and CNTs buckypaper at the frequency range of 1–200 Hz. CNT buckypaper (BP) [29]; SWCNT-BP/epoxy (EP) [27]; MWCNT-BP/EP [75]; SWCNT-BP/poly(ether ether ketone) (PEEK) [76]; MWCNT/polycarbonate (PC) [77]; SWCNT/PC [78]; SWCNT/polyamide 6 (PA6)/poly(methyl methacrylate) (PMMA) [79]; interlocked CNTs at frequency [80]; polyurethane (PU)/EP IPNs [81]; PU/unsaturated polyester (UP) IPNs [82]; nitrile rubber (NBR)/PMMA IPNs [83]; PU/poly(ethyl methacrylate) (PEMA) IPNs [84].

The different microstructures of buckypapers can great affect their mechanical properties, such as viscoelasticity. For buckypapers with long and entangled SWCNTs, the energy dissipates through the zipping-unzipping mechanism during cyclic shearing loading. Such a zipping-unzipping mechanism is found to be highly related to the number of inter-tube entanglements [30]. Therefore, the viscoelasticity of buckypapers with long SWCNTs is both frequency and temperature independent. Unlike the zipping-unzipping mechanism, a sliding-friction mechanism is found to dominate the energy dissipation for buckypapers with short SWCNTs. Although the viscoelastic characteristics of these buckypapers are still frequency independent, their viscosity monotonically increases with increasing temperature. In addition, the loss tangent tanδ linearly increases with SWCNT length before the entangled SWCNT network is formed. When the SWCNTs are entangled together, the tanδ is not sensitive to the SWCNT length anymore. Such an interesting phenomenon is determined by the different energy dissipation mechanisms for buckypapers with different SWCNT lengths. In Figure 10, we compare the viscoelasticity of SWCNT buckypapers in the present study with experimentally-measured values. There are three major regions in this plot. The buckypaper composites usually demonstrate a high storage modulus and low loss tangent (tanδ<0.1), while the inter-penetrating polymer networks (IPNs) demonstrate a high loss tangent (tanδ>0.3) and a low storage modulus. In between, the interlocked CNT networks can exhibit both a high damping and large storage modulus, due to strong inter-tube junctions. The storage modulus and loss tangent of model SWCNT buckypapers fall into another region in this map: small storage modulus and damping ratio. It is also interesting to see that the loss tangent of SWCNT buckypaper can be easily controlled in a broad range upon changing the SWCNT length and the temperature. In the present study, the SWCNT length of each buckypaper is a constant number. It will be interesting to see how the viscoelasticity of buckypaper can be affected by blending short and long SWCNTs together. Moreover, the storage modulus of SWCNT buckypapers can be further enlarged through the densification [72] or inter-tube binding [32].

The obstruction factor β for diffusion of structureless particles in SWCNTs buckypapers is found to be linearly dependent on the volume fraction of SWCNTs ϕ. Note that the volume fraction ϕ changes from a relative large value around 0.5 for short SWCNTs to a very small one of 0.005 for long SWCNTs. As revealed in previous studies, the diffusion behavior of gas or liquid molecules is highly related to the membrane pore size, pore shape and the tortuosity of the flow path [22,37,38]. The pore size of model SWCNT buckypaper varies from nearly zero to 16 nm, as shown in Figure 3. All of these results indicate that the linear scaling relationship between the obstruction factor β and volume fraction ϕ is valid over a broad range of volume fractions and pore sizes, which might be generalized to other fibril networks.

## 5. Conclusions

In this work, the structure-property relationship has been revealed for buckypapers by considering different (5,5) SWCNT lengths. The microstructure and viscoelasticity of buckypaper are found to be dramatically affected by the SWCNT length. When the SWCNT is short, they tend to form bundles together without obvious bending and entanglement. These short SWCNTs are tightly packed together with small pore sizes. The long SWCNT, on the other hand, are more easily bent and entangle together, leaving space for large-sized pores. Due to the different microstructures, the energy dissipation of buckypaper during cyclic shearing loading is dominated by different mechanisms. The sliding-friction and zipping-unzipping mechanisms are found to be responsible for the viscosity of buckypapers with short and long SWCNTs, respectively. The critical length for the transition from a bundle-dominated structure to an entanglement-dominated structure is relevant to the entanglement length of (5,5) SWCNT $L_{e}=100$ nm. Below this critical length, the damping ratio of buckypaper linearly increases with SWCNT length, while the damping ratio is unaffected by SWCNT length, if it exceeds the critical length. Besides, our simulation results uncover that the obstruction factor of buckypapers linearly depends on the volume fraction of SWCNTs, regardless of the dramatic microstructural changes. These obtained results are expected to inspire the future design of novel buckypaper-based materials and enable their applications.
